# High Temperature Growth of Graphene from Cobalt Volume: Effect on Structural Properties

**DOI:** 10.3390/ma11020257

**Published:** 2018-02-07

**Authors:** Giampiero Amato

**Affiliations:** 1 The Quantum Research Laboratory, INRIM, Strada delle Cacce 91, 10135 Torino, Italy; giampiero.amato@uniupo.it; Tel.: +39-011-0437-903; 2 Department of Science and Technological Innovation, University of Eastern Piedmont ‘‘A. Avogadro’’, Viale T. Michel 11, 15121 Alessandria, Italy

**Keywords:** graphene, carbon precipitation, Raman spectroscopy, strain

## Abstract

Several transition metals other than the largely used Cu and Ni can be, in principle, employed to catalyze carbon precursors for the chemical vapor deposition of graphene, because the thermodynamics of their alloying with carbon is well known. For example, the wealth of information in the Co-C phase diagram can be used to predict the properties of graphene grown in this way. It is, in fact, expected that growth occurs at a temperature higher than in Ni, with beneficial consequences to the mechanical and electronic properties of the final product. In this work, the growth of graphene onto Co film is presented together with an extensive Raman characterization of the structural properties of the material so far obtained. Previous results reporting the full coverage with negligible defective areas, in spite of discontinuities in the underlying metal, are confirmed, together with the occurrence of strain in the graphene sheet. Strain is deeply investigated in this work, in view of possible employment in engineering the material properties. The observed strain is ascribed to the high thermal mismatch with the substrate, even if an effect of the crystallographic transition of Co cannot be excluded.

## 1. Introduction

The thermodynamic approach has been largely employed in the study of the different techniques for the growth of innovative materials, since the birth of material science.

Thermodynamic potentials, like the Gibbs’ free energy *G* = *H* − *TS* (*H* being the enthalpy, *S* the entropy, and *T* the absolute temperature), and its derivative with respect to the variation of the quantity of matter, the so-called chemical potential *M* = *δG*/*δn*, allow discriminating between ordered or disordered growth, or by crystalline or epitaxial processes. This approach, however, had no great success in the case of the growth of graphene onto catalyzing metals by chemical vapor deposition (CVD), probably because of the great complexity of this mechanism.

The most used catalyzing metals employed in the CVD of graphene are Ni and Cu. Copper has the lowest affinity to carbon, in fact, it does not form any carbide phases [1], and has very low carbon solubility compared to Co. and Ni. The low reactivity with carbon of Cu can be attributed to the symmetrical electron distribution of the 3d-electron shell {[Ar]3d^10^4s^1^}, which minimizes reciprocal repulsions. The 3d^7^ and 3d^8^ orbitals of Co. and Ni are between the most unstable electronic configuration (Fe) and the most stable one (Cu). Based on this, several investigators considered Cu and Ni as the most suitable catalysts for graphitic carbon formation, whereas Co received less attention [2,3,4,5,6], even if it is a largely employed catalyzer for the growth of C nanotubes. 

**Figure 1:**
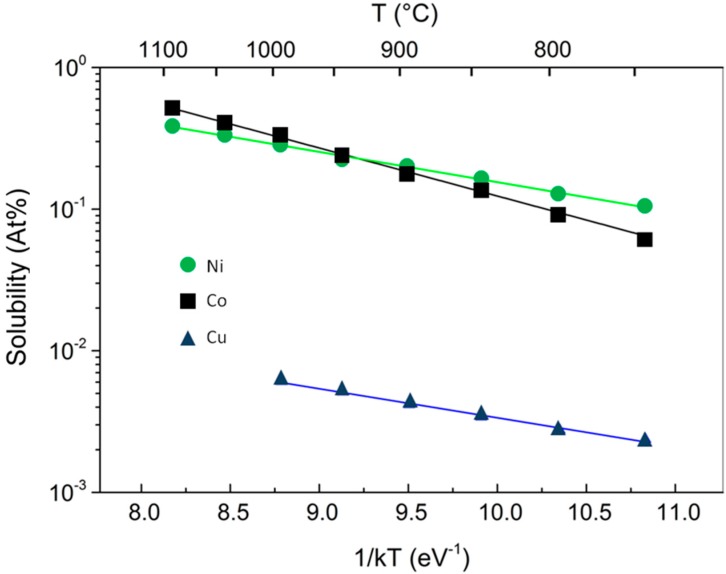
The dependence of the C solubility on the inverse temperature for Co (squares), Ni (circles), and Cu (triangles).

Tracking carbon during the growth process by means of isotope labeling in conjunction with Raman spectroscopic mapping [7,8], demonstrated the different kinetic behavior of CVD growth of graphene on Ni and Cu. The two mechanisms of graphene growth observed on Ni and Cu can be understood in terms of the C-metal binary phase diagram, the most important difference being that, thanks to the much lower solubility in Cu with respect to Ni, only a small amount of carbon can be dissolved in Cu (Figure 1). 

Continuously imaging the C monolayer coverage on Cu using low-energy electron microscopy confirmed no C precipitation or island growth during cooling [9], suggesting that the growth is confined to the surface [10].

In contrast, Co and Ni can dissolve much more C atoms. The graphene growth comes also from the precipitation during the cool-down of the process and “polygraphene” was detected in most cases [10], as a consequence of an excess of precipitation of C atoms. For Co and Ni, the solubility and precipitation process must be controlled to some extent with the annealing, and the isothermal growing and cooling rates. In these cases, we are dealing with a volume growth.

From another point of view, cooling can be considered as an additional way to reduce the entropy of the system. In fact, after the isothermal stage of exposure to hydrocarbons, the entropy of vacancies in Cu-grown graphene can be reduced only by their diffusion during cooling, since no additional atoms are available to saturate them [9,10]. It can be minimized if, e.g., two vacancies collapse into a di-vacancy or by their motion towards the grain boundaries, which act as *vacancy sinks*. In the case of Co and Ni, on the other hand, an extra, effective mechanism for vacancy reduction is provided by the C atoms precipitating from the substrate [11]. 

According to the definition of the Gibbs’ free energy, *G = H* − *TS*, it is straightforward that *G* is minimized with a low-entropy contribution when the deposition temperature is high. It is then advisable that most of the graphene growth occurs during the first stages of cooling. Baraton et al. [12], support this reasoning by reporting about two different growth mechanisms of graphene on Ni. At higher *T*, a lateral growth of high quality graphene occurs at surface curvatures of the catalyzer, and assisted by a diffusive flux of C atoms, whereas, when decreasing *T*, surface precipitation of C occurs, giving rise to nanocrystalline graphene with high entropy.

From a microscopic point of view, the kinetics of precipitation is influenced by the diffusivity of C atoms, which has been reported by Lander et al. [13] to have an activated dependence on ***T*** in the case of Ni:

(1) $$D=D_{0}\cdot exp\left(-\frac{E_{D}}{k_{B}T}\right)$$

with the pre-exponential factor *D*_0_ = 2.48 cm^2^ s^−1^, the activation energy *E_D_* = 1.74 eV, and *k_B_* is Boltzmann’s constant. Calculation gives a diffusion length for C atoms in Ni of the order of few μm in the first 1 s of the cooling stage. This means that C atoms can freely redistribute in the growing film to reduce the entropy of the system. 

Another aspect is the control of precipitation of C atoms onto the surface of the catalyzing metal. If their number is too large, polygraphene is formed. There is intrinsic difficulty in theoretically describing the precipitation of foreign atoms [14] in phase diagrams, because of the presence of different competing mechanisms. The best known are the bulk and surface segregation. While precipitation consists of the phase separation phenomenon in agreement with the thermodynamics phase diagram, equilibrium segregation is a compositional heterogeneity due to either the more rapid cooling of the surface which traps C atoms in the bulk, or to lattice discontinuities of the hosting metal (e.g., internal surfaces at grain boundaries). The quality of the graphene grown in this way comes from a competition of precipitation from the saturated solid solution and bulk/surface segregation. A common recipe, particularly suited to reduce the excess of bulk precipitation, is to employ fast cooling rates even if more sophisticated approaches have also been proposed [15]. The surface micro-segregation is, however, more difficult to control, because the microstructure of the metal plays a role. For example, different deposition methods, or the coalescence of metal grains during heating can give different results. The excess of precipitation occurring at the grain boundaries of the metal substrate is a consequence for this effect and is difficult to avoid. 

The steeper dependence on *T* of the C solubility σ into the metallic substrate, depicted in Figure 1 where Co and Ni are compared, has important consequences on the growth of graphene. The activated behavior for the C solubility is the same for both metals under investigation:

(2) $$\sigma =\beta_{0}\cdot \;exp\left[\frac{-\Delta }{k_{B}T}\right]$$

where *Δ* is the heat of precipitation and *β*_0_ is an entropic pre-factor related to the density of sites into the metal able to host the solute C atoms. Lander et al. [13], experimentally derived these two quantities in case of Ni. Their values, after conversion into atomic concentration and eV, are: *Δ* = 0.42 eV, *β*_0_ = 5.33 × 10^22^ atoms cm^−3^. It has to be noted, in passing, that the value for the heat of precipitation of Cu is comparable, but pre-factor is 2 orders of magnitude lower. The data for Co from the fits in Figure 1 are *Δ* = 0.791 eV, and *β*_0_= 9.06 × 10^23^ atoms cm^−3^, meaning that Co can in principle host ˜10 times more C atoms than Ni, with approximately a double heat of precipitation that makes the T-dependence of solubility more abrupt. This means that in Co, C atoms precipitate at temperature higher than in Ni. This is somehow equivalent to having a much more rapid cooling process, and, according to the discussion above, to grow graphene at higher T, which means increased C atom diffusivity with reduction of the entropy of the growth product.

In the following sections of the paper, several experimental findings on graphene grown on Co will be presented and discussed basing on a simple thermodynamic model. It will be shown how departures from this vision can be explained in terms of other mechanisms, like the different thermal expansion of graphene and substrate. Raman maps of graphene areas, directly taken on the Co growth substrate will be analyzed, with relevance to the structural and mechanical properties of graphene grown in this way.

## 2. Materials and Methods

The mobility of electrical carriers in CVD graphene is known to be limited by scattering with vacancies, grain boundaries, and distorted bonds, even at the nanoscale [16]: such departures from the perfect hexagonal lattice of graphene imply that some atoms vibrate at frequencies different from those of the perfect crystal. This has consequences, e.g., on the Raman response of the material.

The first Raman feature (1590° cm^−1^) of graphene is the well-known ***G*** peak, characteristic for sp^2^-hybridized carbon-carbon bonds. The second prominent feature (≈2690° cm^−1^) originates from a double-resonance process, which creates an electron-hole pair that recombines after two inelastic scattering events involving phonons with opposite momenta and is labeled as the ***2D*** peak. If defects are present, one of the two scattering events can occur elastically and the ***D*** peak, observed in this case, exhibits only half the Raman shift (1350° cm^−1^). Saturated, but distorted, bonds can be present even in case the ***D*** peak is not detected. These sources of entropy have been recently revealed [16] by measuring the ***2D*** linewidth, since it contains valuable information on bond-length variations in graphene in the nanometer range, well below the laser spot size.

The following theoretical treatment of the problem considers, for simplicity, the single atom vacancy as the unique source of entropy of the system, neglecting all the other possible sources of disorder. This allows to easily calculate the variation of the free energy of the graphene layer during sample cooling-down, i.e., when vacancies are saturated by C atoms precipitating from the metal substrate.

The peculiar value for the heat of C precipitation in Co (Δ***_Co._*** = 0.791 eV vs. Δ***_Ni,Cu_*** = 0.484 eV) has been explained by Hasebe et al. [17] in terms of an additional contribution of the internal magnetic field to the Co-C enthalpy. It is worth mentioning that graphene growth is normally carried out at a temperature around 1000 °C, below the Curie temperature *T_C_* of Co, (even if *T_C_* can be somewhat lowered at high concentration of segregated C [17]), but higher than *T**_C_*** of Ni. Thus, the magnetic field contribution is present in Co, but absent in Ni.

The total free energy *G* of a graphene layer in which the vacancies left by the incomplete, isothermal growth have to be annihilated by the successive precipitation step, can be written by adding a term *G*_−1_, the energy for a vacancy annihilation, to the energy balance equation given in [18]:

*G* = *G*_0_ + *nG*_1_ + *G*_2_ − *nG*_−1_ (3)

where *n* is the number of vacancies, *G*_0_ is the free energy of the perfect crystal, *G*_1_ the energy needed to generate one vacancy, and *G*_2_ = −*T*·*S*_c_ (*S*_c_ being the configurational entropy of *n* vacancies). 

The calculations following by imposing the equilibrium condition (*δG/δn = 0*) are reported in [19] yielding the exponential dependence of the concentration of point vacancies *c_v_* as a function of the entropy variation *ΔS*.

Such vacancy concentration will decrease thanks to C atoms precipitating from the metal substrate according to Equation (2), which gives the *T*-dependence of C solubility in the metal:

*c*_*v*_ = *β exp* (−*Δ*/*k*_*B*_*T*) (4)

by considering that the number of C atoms *missing on surface* should have the same *T*-dependence of the number of C atoms *diffused underneath* into the metal. In the ideal situation *β* = *β*_0_, Equation (2), but deviations from ideality are obvious, and related to additional effects competing with the C atoms precipitation, segregation above all. Additionally, a further dependence of *β* on the cooling rate cannot be excluded. For sake of simplicity, we will consider *β* as a phenomenological constant, reasonably lower than the pre-exponential factor of C solubility, and related to the availability of C atoms dissolved into substrate, obtaining [19]:

*ΔS* = −(*k*_*B*_*ln* (*α*/*β*) + *Δ/T*) (5)

Equation (5) provides the reduction of entropy when *one* vacancy is annihilated. This is given by two terms: the first is related to the balance between *α* vacancies and *β* atoms available to precipitate, the second is related to the *rate* of precipitation, which is given by the heat of precipitation *Δ*. It is evident that in the growth (*β* < *α*), both terms are positive in sign and the total entropy variation *ΔS* is negative, as expected from the annihilation of *one* vacancy. The important point is that a larger value of *Δ* yields the same entropy variation *ΔS*, but at higher *T*, which means a larger reduction of *G*.

The experiment consisted in synthesizing graphene via CVD, using CH_4_ as a precursor gas either in a chamber of a rapid thermal processing (RTP) apparatus [20], or in a furnace with a quartz tube. 500 nm thick, Co polycrystalline thin films were deposited either by RF sputtering (100 W RF power, 10^−2^ mbar Ar^+^ pressure, deposition rate around 3 Å/s) or by thermal evaporation onto Si substrates with 500 nm thick SiO_2_. Substrates, sonicated in acetone and ethanol and then inserted in the reactor chamber, were exposed to a H_2_ atmosphere for 5 min at 1000 °C, to remove the residual Co oxide, then 10 standard cubic centimeters per minute (SCCM) of CH_4_ and 20 SCCM of H_2_ were delivered for 5 min at the same temperature and a pressure of 6.7 mbar. Cooling was carried out in two ways: by switching the lamps off in the RTA system or by sliding the sample out of the heated zone in the tubular furnace. In both cases a flux of few hundreds SCCM inert gas (Ar/N_2_) was delivered into the chamber. The cooling rates of 3.5 °C/s and 10 °C/s have been estimated in the RTP system and in the conventional tube, respectively. In a previous publication, we have shown that our Co-grown graphene can be transferred by means of the well-known wet method consisting of immersing the sample into a metal etchant and picking up the floating graphene flake by means of the destination substrate [21]. This procedure previously requires to strengthen the graphene layer by spinning a polymer onto it. This can be avoided in Co-grown graphene, and the self-standing membrane can be transferred without any contact with a polymer. As explained in [21], the commonly employed poly-methyl-methacrylate (PMMA) is a p-type dopant, then, thorough cleaning is required in the following electrical characterization. Such a problem has been avoided in our case, since we decided to totally eliminate the PMMA by structuring the field effect devices by evaporating the metal contacts through a mechanical mask. In this way, mobility values of the order of 10^4^ V cm^2^ s^−1^ have been measured in a large sample portion displaying typical monolayer characteristics [21]. 

Micro-Raman analysis has been performed in a homemade system [21,22]. All acquisitions of Raman spectra were performed with a laser (λ = 532 nm, wavelength) spot diameter of 1 µm and power of *P* ≈ 2 mW. The spectra were collected at power density *Ψ* ≈ 6.4 × 10^4^ W/cm^2^ and acquisition time *t*_acq_ = 60 s. 

While copper emits strong photoluminescence when irradiated with green light, so hindering reliable Raman measurement on the as-grown layer, cobalt is optically inert in the typical wavelength range of dispersive Raman spectroscopes. This allows the ability to extract information about the properties on the as-grown layer and on the possible effect of the transfer step. In [21], a careful study of the PMMA-free transfer method described above is presented, based on a statistical analysis of large graphene areas mapped by Raman spectroscopy. 

The final result is that defects provoked by our transfer method of Co-grown graphene are less than those induced when transferring the Cu-grown, even when using the supporting PMMA layer.

Light scattered from the sample has been analyzed by means of a circular polarizer (Edmund Optics, Barrington, NJ, USA) placed at the entrance of the optical fiber that delivers the emission to the monochromator. It selects a linearly polarized component of the light emitted by the sample, and then through a quarter-wave plate, polarizes it circularly, avoiding any accidental intensity attenuation due to the interaction with the fiber, or the monochromator optics. The (vertical) polarization of the pump laser beam has been kept fixed to avoid the same kind of problem with the microscope optics. Polarized Raman analysis has then been performed by rotating the analyzer, and, if necessary, the sample under the microscope (Leica Microsystems, Wetzlar, Germany).

## 3. Results and Discussion

**Figure 2:**
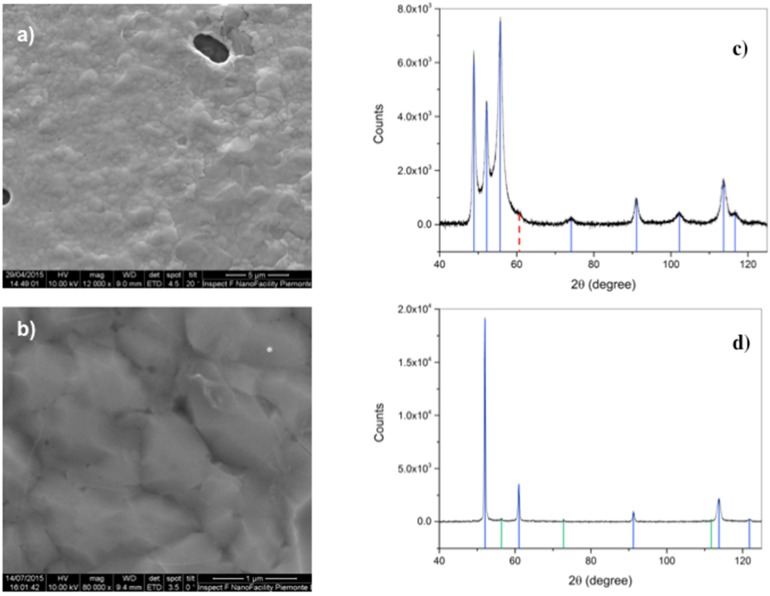
The surface morphology of the Co films after graphene deposition, observed by scanning electron microscopy at different magnifications, and revealing: (**a**) the formation of circular/elliptical holes in the metal, that in RTP (rapid thermal processing) samples are covered by graphene; and (**b**) the wrinkles of the graphene layer lying on the rough surface of Co. In (**c**) the X-ray diffraction spectra of the as deposited Co film, in which the Co hcp peaks are identified (blue lines), as well as the negligible fcc phase (dotted red line). In (**d**), the same for Co. after deposition where the Co fcc peaks are identified by blue lines. Green lines indicate Co silicide peaks.

After deposition, scanning electron microscopy has been performed as a first morphological characterization step, evidencing the presence of circular/elliptical holes in the metal substrate, due to dewetting (Figure 2). The coverage of the Co film in samples deposited by means of RTP overcomes 95%, whereas it is less than 90% in the case of deposition in the conventional quartz furnace. This suggests that the duration of the heating step plays a role [23], since in the first case temperature is raised at a rate of 5 °C/s, due to instrumental limitations, and with a rate 10 times lower in the second. Another important difference is that we observe suspended graphene on the metal discontinuities in the RTP samples, and not in the ones processed in the quartz tube. The situation is similar to that reported for Cu films [20,23], despite the melting T of Co is about 400 °C higher than Cu. 

The preliminary conclusion about the mechanism of graphene growth is that it starts during the isothermal exposure to CH_4_. In fact, in this step, the holes in the metal are still in their incubation stage [24,25] in the RTP process, while during the longer heating in the conventional furnace they are already formed. Hence, the graphene growth is hindered in this last case. The scenario then considers two competing mechanisms taking place in RTP during the isothermal exposure to the precursor, graphene growth and film dewetting. Carbon precipitation on the metal surface obstacles dewetting [23], which is on the contrary favored by Co-C alloying at the high temperature (T = 1000 °C) involved.

Another important aspect to be taken into account is the crystallographic transition, occurring in Co at temperatures around 420 °C. As displayed in Figure 2c,d, the initial hcp phase of Co is replaced by the fcc, then, possible strain at the microscopic scale has been already suggested to be induced in graphene by this transition occurring during cool-down [6]. 

Recently, Pudikov et al. [26] described an interesting experiment in which C atoms coming from the substrate of HOPG (highly-oriented pyrolytic graphite), precipitate (these authors, together with several others, use the term “segregate” instead of “precipitate”, adopted here) under cooling on the surface of a thin metal (Co, Ni) film. They report about a better ordered surface of graphene grown on Co/HOPG, confirming the expectations from the thermodynamic model reported in [19]. This result, together with the reported better mechanical stability and encouraging electrical properties [21], contributes to establishing a picture in which the film entropy is lower than in Ni thanks to the occurrence of C precipitation at higher temperatures. Entropy in such graphene layers is no longer related to lattice discontinuities (defects or domain boundaries), as in Cu-grown grapheme; rather, it is due to bond deformations, excess of precipitation, or strain. Materials efficiently relieve strain through fracture; in other words, a further reduction of ***G*** occurs by means of the increase of *S* consequent to the break of some atomic bonds. At these strain levels this phenomenon has not been observed, even in the PMMA-free transfer, and strain has been reported also on the destination substrate [21]. This observation is not possible in Cu-grown graphene for two reasons, namely, the occurrence of fractures after transfer [10], and the intrinsic difficulty for most investigators to apply the Raman technique on samples still on luminescent Cu. Such analysis is then crucial to ascertain the origin of strain, its effect on the electrical transport properties, or its possible applications, e.g., to tailor the density of states (DOS) of graphene. Then, in the following, we will focus onto this analysis to discriminate between isotropic or uniaxial, tensile or compressive, strain in the samples under study. 

### 3.1. Unpolarized Raman Analysis

**Figure 3:**
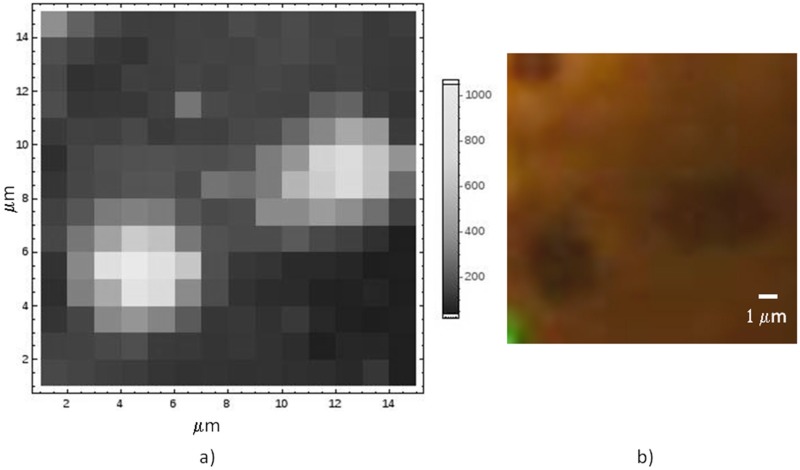
Raman analysis of graphene grown as suspended on metal holes: (**a**) the intensity of the Raman emission of graphene at ~2690 cm^−1^, the so-called ***2D*** peak; and (**b**) the corresponding optical image displaying the three holes and the laser spot.

A typical Raman map on graphene still lying onto its Co substrate is reported in Figure 3, together with the optical image, displaying discontinuities in the metal as darker spots. The enhancement of the Raman signal in correspondence of the metal holes is related to the interference effect, similar to what normally used when graphene is transferred onto oxidized Si [27].

The enhancement of the signal in correspondence of the substrate holes does not allow to directly measure of the number of layers by means of the ***2D/G*** intensity ratio. The reason lies on the fact that the difference in wavelength of the two peaks is 40 nm, and the interference effect can greatly change their relative intensities [28]. On the contrary, the ***D*** and ***G*** peaks are only 8 nm far, then, that ratio can still work for measuring the defect content of graphene. 

On the other hand, the optical enhancement allows for very sensitive measurements on the suspended areas. In a previous paper [21], we reported about the observation of tensile strain in the suspended graphene layer which somehow propagates to the neighboring areas. A signature of such tensile strain is the redshift of the main Raman modes, ***G*** and ***2D*** as a consequence for the weakening of bonds and the decrease of the vibration frequency. In the case of uniaxial strain, however, the deformation of bonds along perpendicular directions will be opposite: this leads to a splitting of the Raman modes, in particular, the double-resonant one, since it involves transitions between two Dirac cones [29,30]. ***2D*** phonon broadening and softening is then detected in strained graphene, but it can be somehow confused with the presence of multilayered graphene. To shed more light onto this phenomenon, the Raman analysis has been applied to several holes with different shape and dimension. In fact, the simple model proposed in [21] suggests the strain in the graphene layer to be a consequence for its different expansion with respect to the substrate during cooling.

The negative thermal expansion coefficient in graphene has been reported to be of the order of 10^−5^ K^−1^ at room T [31,32]. In a similar way, the hole in the underlying metal expands during cooling: its expansion coefficient is negative, too, and can be calculated [21] as *γ =* −*λ_Co_. x_o_/d*_0_, where *λ_Co_* = 1.6 10^−5^ K^−1^ is the thermal expansion coefficient of the Co film, *x_o_* is a length somehow related to the average distance between holes, and *d*_0_ is the hole’s dimension. We expect that smaller holes expand more than graphene when cooling, producing tensile stress, whereas for larger hole diameters, the opposite happens and compressive stress is applied. The dimension of the hole at the crosspoint heavily depends on the parameter *x_o_*, which is quite arbitrary, and an experiment to confirm this effect is necessary. 

**Figure 4:**
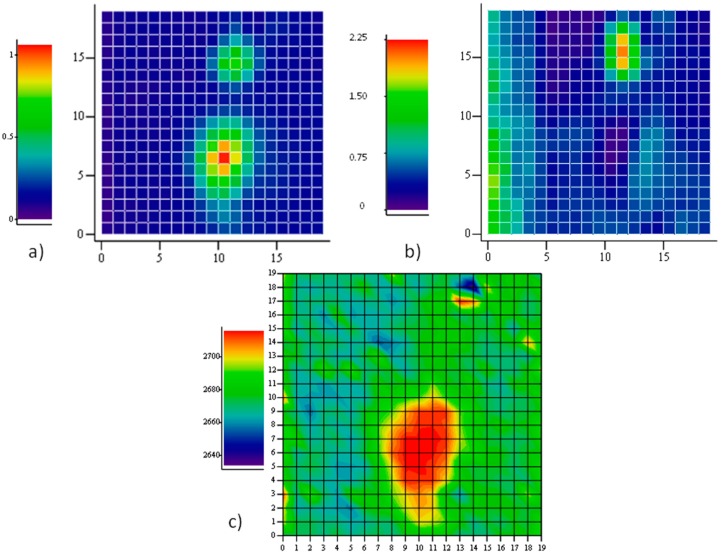
Raman map on a 20 μm × 20 μm area of graphene grown on Co: (**a**) the intensity of the Raman emission of graphene at ~2690 cm^−1^, which allows to localize two holes in the underlying metal. The Raman intensity is normalized to 1 at maximum; (**b**) the ratio between the intensity of the defect peak D and the G peak; and (**c**) the map of the centroid of the 2D feature, indicating a large shift towards higher energies in correspondence of the larger hole in Co, whose major axis approaches 10 μm.

Figure 4 reports a Raman map taken on a region, 20 μm × 20 μm, with one hole with a major axis close to 10 μm. The intensity map of one of the main Raman modes (***2D*** in this case) allows the recognition of holes in the film underneath, as described. When mapping the position of the ***2D*** mode, however, a large shift towards higher energies is observed on the large hole, indicating the presence of a large compressive strain therein. Our apparatus measures the ***2D*** mode of unstrained graphene grown on Cu and transferred onto SiO_2_ to be at 2684 cm^−1^. Hence, a shift of several tens of cm^−1^ is observed in this case. The map of the intensity ratio between the ***D*** and the ***G*** modes does not report on the presence of defects in the graphene portion onto the large hole, rather, a large fragmentation is observed through an exceptional intensity of the ***D*** mode onto the other, smaller hole at coordinates (13,17). The fact that no strain is detected here suggests that some fracture occurred, caused by the intense (maybe tensile) stress provoked by the expansion of the smaller hole. On the larger one, instead, compressive stress takes place, in agreement with the predictions of our model based on the different thermal expansion of graphene and its substrate.

As mentioned before, both the two main Raman features of graphene, ***G*** and ***2D***, undergo splitting, as a consequence for the application of stress. Bonds oriented in different directions can become longer or shorter, then vary their frequency accordingly. The so-called ***G***^+^ and ***G***, or ***2D***^+^ and ***2D***^−^ features will then appear in the Raman spectra, with consequent broadening and softening of the peaks. Experimentally, a larger splitting is observed in the case of the ***2D*** mode, and this is the reason why we deeply investigated this feature. In fact, a large splitting is, in principle, observable only in the case of uniaxial stress: when subjected to isotropic stress, the difference of the bond lengths is only due to differences of the Young moduli along the principal directions, armchair and zigzag. 

Our samples show typical isotropic strain, then, the fair splitting of the ***G*** feature does not always allow for a reliable deconvolution of its components, which is possible, instead, in the case of the ***2D*** mode. It cannot be excluded, however, that some uniaxial strain takes place along some preferential directions. The polarized Raman analysis has been carried out reveal the presence of such uniaxial components. 

### 3.2. Polarized Raman

The fit of the two Lorentzian components of the Raman modes has been carried out by means of the Levenberg-Marquard method, combined with a Monte-Carlo routine for randomly selecting the starting values of the fitting parameters. 

**Figure 5:**
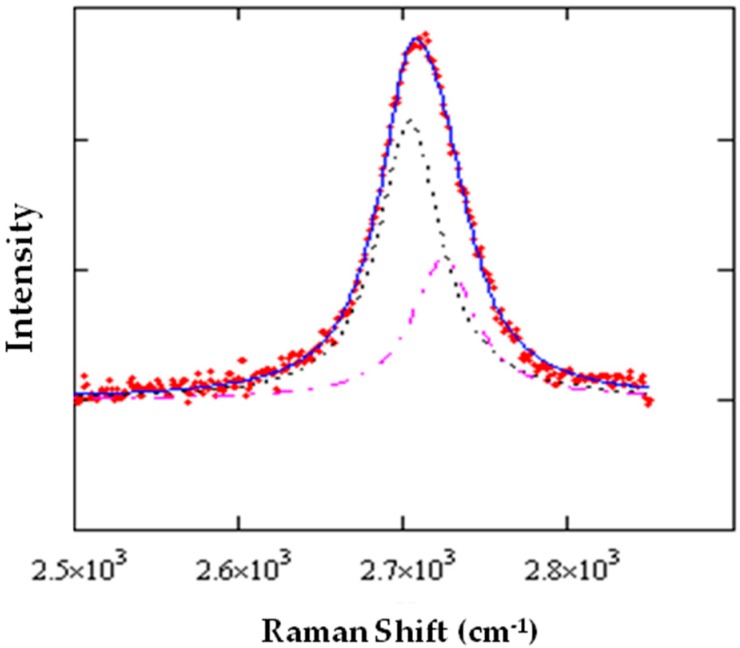
Typical ***2D*** splitting of strained graphene grown on Co: the full line is the fit of the experimental points obtained by summing the two Lorentzian peaks (dotted and dash-dotted lines) representing the ***2D***^−^ and ***2D***^+^ components, respectively.

A typical result is displayed in Figure 5. It can be seen that at the large blue-shift of the ***2D*** peak corresponds to a relatively small splitting, an observation consistent with the presence of isotropic strain. This analysis has been applied to spectra taken on the same spot (generally, a graphene area suspended onto a hole) at different rotation angles of the polarization analyzer, from 0° to 360°.

In another approach, polarized Raman maps have been collected at fixed analyzer positions.

The full characterization of strain in materials requires the knowledge of almost three among the direction of polarization of the incident and emitted light, the direction of uniaxial strain, and one well-defined crystallographic direction (normally, the armchair direction is considered as the x axis in graphene [30]). In the case under study, only the first two angles are known. By analyzing the laser light reflected by a mirror placed in the sample position, we were able to fix the 0° direction of collected light as coinciding with the direction of polarization of the incident beam. Different angles of scattered light can be then ascribed to strain, while nothing can be said about the crystallographic direction, as the sample is polycrystalline. 

**Figure 6:**
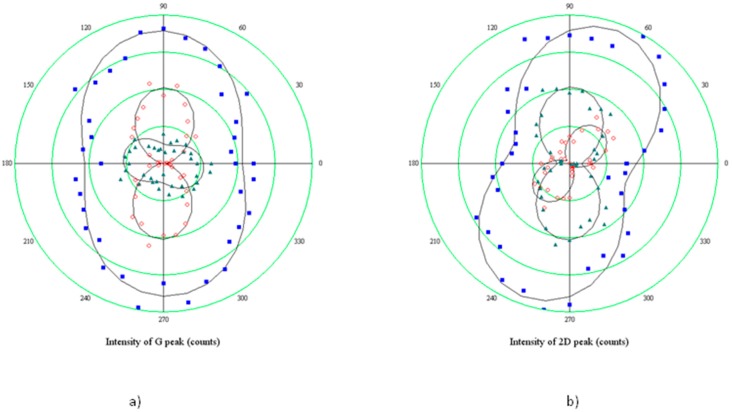
Angular dependence of components of the split peaks in strained graphene grown on Co: (**a**) polar plot of the integrated intensity of the Raman emission of graphene at ~1590 cm^−1^ (***G*** mode) as a function of the angle between the direction of polarization of the incident beam and the analyzer; circlets refer to ***G***^−^, triangles to the ***G***^+^, dots to the total intensity, lines are sinusoidal fits; (**b**) The same as in (**a**), but for the ***2D*** peak. The orientation of the sample is the same for both graphs, with the minor axis of the elliptical hole oriented parallel to the polarization direction of the laser beam.

The angular dependence of polarized light has been measured at intervals of 10°, by manually rotating the analyzer. In the case shown in Figure 6, it was possible to reliably measure and deconvolute either the ***G*** or the ***2D*** peaks. It can be observed that, in the first case, the two components have a phase difference of roughly 90°, which is consistent with the evidence that the linear polarization of radiation diffused by the two phonon modes are orthogonal, independently if ***G***^−^ (***G***^+^) is oriented along the armchair (zigzag) direction or the opposite.

**Figure 7:**
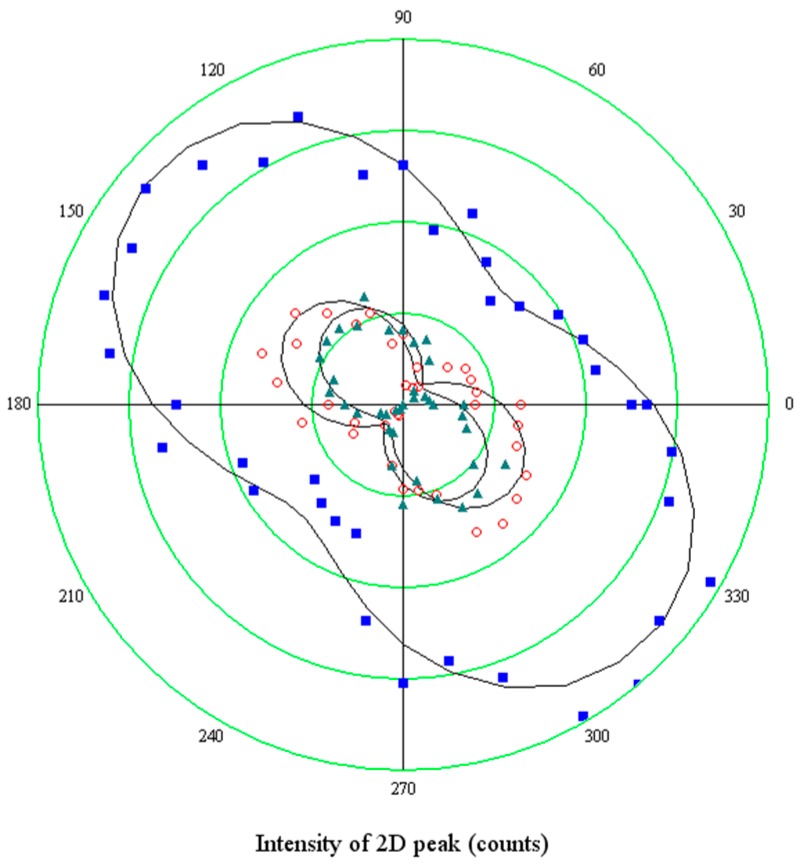
The same angular dependence of components of the split ***2D*** peak shown in Figure 6b, but with the hole minor axis rotated perpendicular to the polarization axis of the laser beam.

The polar plot in Figure 7 reports the results obtained when rotating the sample of 90° with respect to the polarization direction of the incident light (coinciding with the 0° direction of the analyzer). Hence, the minor axis of the elliptical hole is rotated perpendicular to the optical system.

The two components show a rather different behavior: ***2D***^+^ rotates of about 90° coherently with the optical reference system, clearly indicating no variations of the polarizability tensor, which, on the contrary, varies for the ***2D***^−^ component, since it rotates 50°, and the intensity of the mode becomes comparable with the ***2D***^+^. It could be concluded that shortened bonds, which are responsible for the higher energy ***2D***^+^ sideband, do not have any preferential direction of vibration, while the vibration of stretched bonds indicates a preferential alignment of tensile strain along the hole minor axis, in agreement with the model which relates the build-up of the tensile stress to the expansion of small holes.

**Figure 8:**
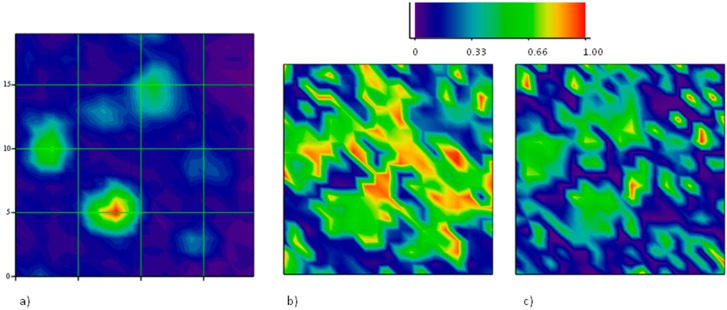
Polarized Raman mapping of strained graphene grown on Co: (**a**) integrated intensity map of the ***G*** mode which allows to localize six different graphene regions suspended onto holes; (**b**) the same as in (**a**), but for the ***2D***^−^ peak, normalized onto the total ***2D*** intensity. In this case the analyzer is oriented parallel to the polarization direction of the incident laser; (**c**) the same as in (**b**), but with the analyzer oriented orthogonal to the polarization direction of the incident laser. The orientation of the sample is the same for both polarized maps (**b**,**c**).

The Raman mapping of the two components of the ***2D*** feature have been carried out at different directions of polarization of the scattered light. A sample area of 20 μm × 20 μm with several holes has been selected. In the intensity map of the region, 6 holes have been localized. In Figure 8, they are identified as an increase of the intensity of the ***G*** peak. The z-axis is normalized to the maximum intensity value, so, the different colors of the holes truly represent the different peak amplitudes. This is not related to some spatial variations of the structural properties of the graphene sheet, but rather to different depths of the holes beneath, which tune the interference enhancement of the Raman signal. 

The two differently polarized maps have been extracted by rotating the analyzer parallel and perpendicular to the polarization of the incident laser beam without changing the orientation of the sample with respect to the optical system. Spectra of the ***2D*** peaks have been collected and analyzed according to the method described above. The results displayed in Figure 8 report the spatial distribution of the integrated intensity of the ***2D***^−^ component, normalized to the integral of the whole peak. Then, the spatial distribution of ***2D***^+^ can be inferred as complementary. 

It is shown that all the graphene regions onto holes of this area undergo tensile strain, since the low energy sideband of the ***2D*** mode dominates. It is interesting to observe that the prevalence of the ***2D***^−^ mode occurs in the intermediate region in between the holes (see panel b), as a composition of forces originating in the holes. However, when an orthogonal polarization state for the emitted radiation is chosen (panel c), the intensity of the ***2D***^−^ mode is quenched in the area between the holes. This is a clear indication that strain is uniaxial there. 

A question that may arise is why graphene grown on Ni does not show strain at this level, since the thermal expansion coefficient of Ni differs of only a few % only from that of Co. The answer can be given by applying the law of thermal expansion to two two-dimensional solids. Assuming the two solids with different expansion coefficients ***k*_1_** and ***k*_2_** have the same surface *S*_1_ *= S*_2_ at *T_h_ > T_l_*, when their temperature is decreased to *T_l_*, their surface areas will be *S*_1*′*_ *≈ S*_1_*(1* − *2k*_1_Δ*T)*, *S*_2*′*_ *≈ S*_2_*(1* − *2k*_2_Δ*T)*, and their difference in surface will be |Δ*S| ≈ 2|S*_1_*k*_1_ − *S*_2_*k*_2_*|*Δ*T*, proportional to the temperature step. This is what happens to the Co-graphene system, which is formed at *T* higher than in Ni.

In the previous section, we mentioned about another possible origin for strain in graphene. A possible role of the crystallographic transition occurring in Co at temperature around 450 °C from fcc to hcp has already been suggested [6]. This is, however, not trivial to observe, because the Co film is polycrystalline, with grains having different orientations at the surface. It can be recalled, inter alia, that Co fcc cells, with <111> lattice plans at the surface, show hexagonal structure with dimension close to that of graphene. In that case, the lattice of the overlying graphene can be perturbed by departures from the fcc structure of Co, while one should expect that this occurs in the case of the opposite transition, from hcp to fcc. Hence, the hydrostatic strain component that can be observed as compressive could be speculated to arise from such kinds of crystallographic transitions of the substrate.

## 4. Conclusions

A drawback of the volume growth of graphene with respect to the case of Cu is the difficulty of controlling precipitation along the cooling stage. From this point of view, Co offers the possibility of growing graphene at higher temperatures, reducing precipitation at moderate temperatures. This is due to the larger heat of precipitation of C atoms in Co. The thermodynamic treatment suggests the reduction of entropy in the graphene sheets grown in this way with respect the case of Ni, while recent results [21,26] confirm superior mechanical stability and encouraging electrical performances of this material as a consequence of the better ordered structure. Strain is a direct consequence of the growth mechanism and its role can be two-fold: if lattice deformations at the nanoscale are detrimental to the electronic properties [15], strain generated at the metal discontinuities as a consequence for the different thermal expansion coefficients allows, in principle, to engineer the density of states during growth. Even if a quantification of strain is not trivial because we are dealing with a complicated superposition of isotropic and uniaxial components, either tensile or compressive, it can be estimated to be in the percent range [21], a value sufficient to open the energy gap in graphene [29].
